# Endoscopic submucosal dissection for esophageal cancer behind a tracheoesophageal voice prosthesis

**DOI:** 10.1055/a-2254-7503

**Published:** 2024-02-15

**Authors:** Yasuhiro Tani, Koji Higashino, Takashi Kanesaka, Kengo Aoki, Takashi Fujii, Tomoki Michida, Ryu Ishihara

**Affiliations:** 1 Department of Gastrointestinal Oncology, Osaka International Cancer Institute, Osaka, Japan; 2 Department of Gastroenterology and Hepatology, Osaka University Graduate School of Medicine, Suita, Japan; 3 Department of Head and Neck Surgery, Osaka International Cancer Institute, Osaka, Japan

A tracheoesophageal voice prosthesis is used to restore vocal communication after a total laryngectomy 1 2 . This device may interfere with the endoscope during endoscopic treatment of an esophageal lesion. However, its removal poses a risk of aspiration because it is located in a tracheoesophageal fistula. We successfully performed endoscopic submucosal dissection (ESD) for superficial esophageal squamous cell carcinoma behind a tracheoesophageal voice prosthesis.

A 65-year-old man was diagnosed, by means of upper gastrointestinal endoscopy, as having a superficial lesion on his upper thoracic esophagus. The oral part of the lesion was behind a tracheoesophageal voice prosthesis, which was placed after total laryngectomy and jejunal interposition for hypopharyngeal cancer. ESD was planned to be performed under general anesthesia to reduce the risk of aspiration when the tracheoesophageal voice prosthesis was removed.

The patient underwent tracheal intubation, and the intubation balloon was placed caudally to the lesion to prevent aspiration ( Fig. 1 ). After the voice prosthesis was removed, the endoscope was inserted, and markings were made around the lesion ( Video 1 ). ESD was performed using a FlushKnife BT-S (1.5 mm, DK2620J; Fujifilm Medical, Tokyo, Japan) and the lesion was resected en bloc. After replacement of the voice prosthesis, extubation was performed. Histopathological examination revealed squamous cell carcinoma confined to the epithelium. As the lesion was completely removed endoscopically, the patient was followed up without additional treatment. After 8 months, no local recurrence was detected on surveillance endoscopy.

**Fig. 1:**
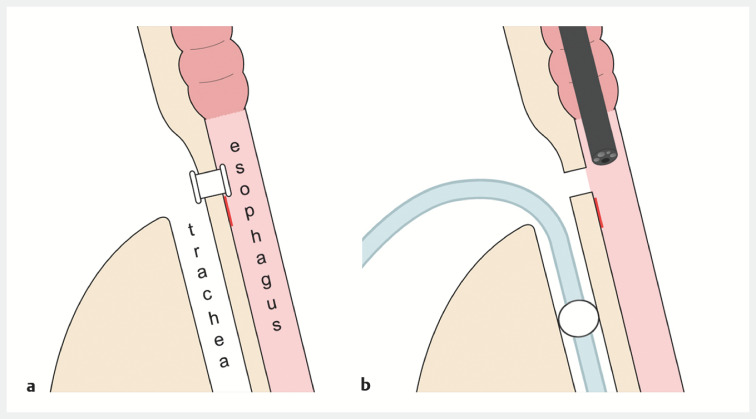
Schematic diagrams. **a** The oral part of the superficial esophageal lesion behind a voice prosthesis. **b** The intubation balloon was placed caudally to the lesion and the voice prosthesis was removed.

Endoscopic submucosal dissection for a superficial esophageal lesion, the oral side of which was behind a tracheoesophageal voice prosthesis.Video 1

ESD was successfully performed without any adverse events under general anesthesia by adjusting the cuff position of the intubation tube.

Endoscopy_UCTN_Code_TTT_1AO_2AG

## Notes

Endoscopy E-Videos https://eref.thieme.de/e-videos
*E-Videos* is an open access online section of the journal *Endoscopy* , reporting on interesting cases and new techniques in gastroenterological endoscopy. All papers include a high-quality video and are published with a Creative Commons CC-BY license. Endoscopy E-Videos qualify for HINARI discounts and waivers and eligibility is automatically checked during the submission process. We grant 100% waivers to articles whose corresponding authors are based in Group A countries and 50% waivers to those who are based in Group B countries as classified by Research4Life (see: https://www.research4life.org/access/eligibility/ ). This section has its own submission website at https://mc.manuscriptcentral.com/e-videos .
